# Bi-Directional Long Short-Term Memory-Based Gait Phase Recognition Method Robust to Directional Variations in Subject’s Gait Progression Using Wearable Inertial Sensor

**DOI:** 10.3390/s24041276

**Published:** 2024-02-17

**Authors:** Haneul Jeon, Donghun Lee

**Affiliations:** Mechanical Engineering Department, Soongsil University, Seoul 06978, Republic of Korea; whtotlfqj@soongsil.ac.kr

**Keywords:** gait phase recognition, time-series data, sliding window, bi-directional LSTM, wearable sensor

## Abstract

Inertial Measurement Unit (IMU) sensor-based gait phase recognition is widely used in medical and biomechanics fields requiring gait data analysis. However, there are several limitations due to the low reproducibility of IMU sensor attachment and the sensor outputs relative to a fixed reference frame. The prediction algorithm may malfunction when the user changes their walking direction. In this paper, we propose a gait phase recognition method robust to user body movements based on a floating body-fixed frame (FBF) and bi-directional long short-term memory (bi-LSTM). Data from four IMU sensors attached to the shanks and feet on both legs of three subjects, collected via the FBF method, are processed through preprocessing and the sliding window label overlapping method before inputting into the bi-LSTM for training. To improve the model’s recognition accuracy, we selected parameters that influence both training and test accuracy. We conducted a sensitivity analysis using a level average analysis of the Taguchi method to identify the optimal combination of parameters. The model, trained with optimal parameters, was validated on a new subject, achieving a high test accuracy of 86.43%.

## 1. Introduction

In human locomotion, walking constitutes a reflexive biomechanical process characterized by repetitive phases, encompassing various physiological and mechanical factors, including muscular activity, neurological control, cutaneous sensation, and kinematic dynamics like force exertion and velocity [1]. Gait data, reflecting distinct physical and mechanical attributes within each phase of the movement, are utilized extensively not only in rehabilitation [2,3], healthcare [4], and medical care [5,6] but also in providing daily assistance [7,8] and support in physical training [9].

In analyzing gait data, the accurate identification and prediction of distinct gait phases [10] are crucial for reliable interpretation and subsequent applications. Commonly, the detection of gait phases employs a variety of sensors, such as wearable Inertial Measurement Unit (IMU) sensors, vision-based sensors, surface Electromyography (sEMG) sensors, and Force-Sensitive Resistor (FSR) sensors. Although motion capture systems, a type of vision sensor, offer high accuracy in gait phase detection, they are susceptible to limitations such as light sensitivity and occlusion and their restricted workspaces [11]. While surface Electromyography (sEMG) sensors can effectively collect muscle activation signals for enhanced intent recognition in gait analysis, they face limitations due to individual variances in body fat and physical condition and require extensive signal processing to address noise-related challenges [12]. FSR has the advantage of having a higher recognition accuracy than other sensors. However, the sensor must be located accurately on each foot to ensure high accuracy, and it has low durability because it is exposed to strong impacts [13,14]. Therefore, an IMU sensor, known for its wearability and reliability, is predominantly used either independently or in conjunction with an FSR as an auxiliary device to augment its capabilities [15]. Zhen et al. [16] achieved a 91.8% accuracy rate in gait phase recognition using a combination of three IMU sensors and the LSTM-DNN algorithm. However, due to the characteristics of IMU sensors, which always represent data relative to a global frame, maintaining a consistent initial posture and orientation is essential to ensure uniform data acquisition. Gait data acquisition and validation were conducted on a treadmill to ensure uniformity and to enhance recognition accuracy; the gait cycle was categorized into two distinct phases: stance and swing. Vu et al. [14] utilized an RNN with one IMU and two FSR sensors to recognize gait phases, achieving consistent gait phase prediction with an average estimation error of only 2.1 ± 0.1%. Similar to the previous study, this research also performed data collection and validation on a treadmill for consistent data values. Moreover, it was noted that there was a variability in error deviation among different subjects, attributed to the limitations of the RNN model.

In studies that integrate IMU and FSR sensors for gait phase recognition, challenges include the IMU sensors’ low reproducibility in attachment and decreased accuracy due to movement [17]. Moreover, the difficulty distinguishing between stance phases, due to minimal variation in sensor data, often leads to poor recognition accuracy. This also results in a generalized classification of gait phases into broad categories like swing and stance, without further subdivision into sub-phases [18,19].

In previous studies on gait phase recognition using IMU sensors or a combination of IMU and FSR sensors, certain limitations commonly arose, which are detailed below:

1. Recognition accuracy was reduced in subsequent experiments due to the low reproducibility of sensor attachment when using IMU sensors.
2. The nature of IMU sensors’ dependency on a global reference frame necessitates maintaining a fixed initial posture and orientation for consistent data gathering.
3. Labeling and differentiating the subtle sub-phases of the stance phase becomes challenging due to minimal variability in IMU sensor outputs.

In this study, to address the identified limitations, the floating body-fixed frame (${FB}_{f}$) concept from previous research was adopted [20]. This approach ensures consistent IMU sensor data collection, irrespective of attachment variability or changes in the wearer’s posture. Additionally, the high phase recognition accuracy of FSR sensors is utilized for more precise gait phase division and labeling, aiding in the differentiation of subtle sub-phases within the stance phase. To enhance the gait phase recognition model’s performance, this study focused on identifying and analyzing the most impactful parameters and hyperparameters. Through a thorough selection process and sensitivity analysis, the optimal parameter set was determined, ultimately leading to higher test accuracy in the model.

## 2. Materials and Methods

### 2.1. Gait Phase Division and Labeling Method

**Figure 1:**
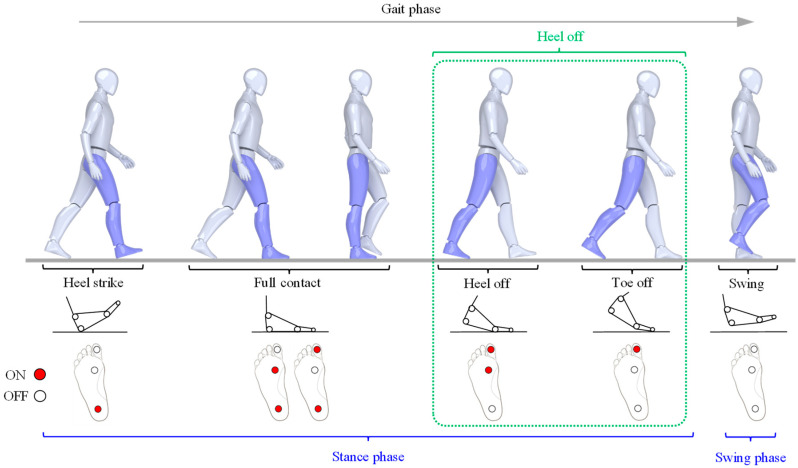
Four gait sub-phases according to 3-ch FSR measurement results.

Generally, in biomechanics and ergonomics, the gait phase is divided into the swing phase (SW) and the stance phase, based on foot–ground contact, as shown in Figure 1. The stance phase is further subdivided into heel strike (HS), full contact (FC), heel off (HO), and toe off (TO) [21]. However, it is observed that as a person’s walking speed increases, the relative duration of the heel-off (HO) and toe-off (TO) phases within the entire gait cycle decreases [22]. In this study, based on previous research that demonstrates the toe-off (TO) phase constitutes only a minor portion (1.59%) of the general gait cycle, TO was merged with the heel-off (HO) phase. Consequently, the gait cycle was divided into four distinct phases: swing (SW, label 5), heel strike (HS, label 4), full contact (FC, label 3), and heel off (HO, label 2) [23].

**Figure 2:**
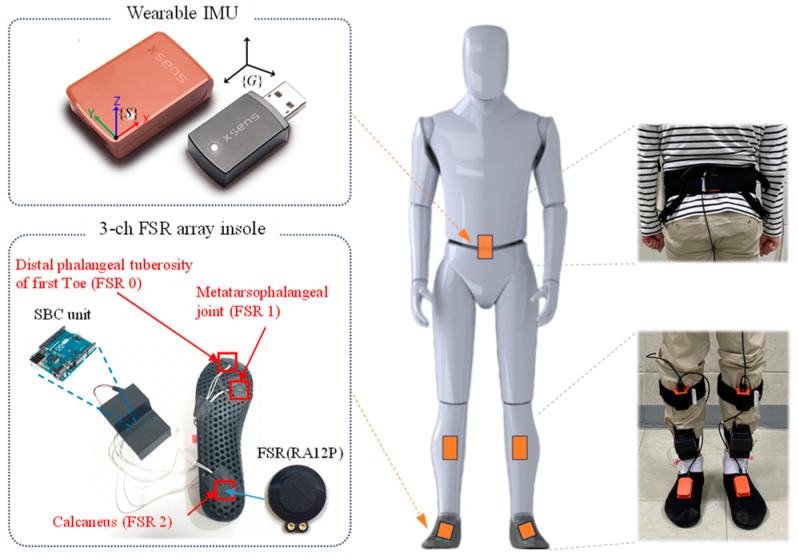
Sensor configuration and attachment location for data acquisition.

To predict the four selected gait phases using IMU and FSR sensors, a measurement unit that can simultaneously label IMU sensor output data in the same measurement cycle is necessary. Figure 2 displays a wireless insole unit equipped with FSR, designed to label gait phases concurrently as IMU sensor data are collected. To collect gait data, IMU sensors are attached using straps at specific body locations: one on the waist, one on the top of each foot, and one on each shank. These sensors capture lower extremity gait data at a sampling rate of 100 Hz. For the FSR insole unit, which labels IMU sensor data, an FSR sensor was placed in three key foot areas: the distal phalangeal tuberosity of the first toe, the metatarsophalangeal joint, and the calcaneus. This configuration is based on the patterns of foot pressure distribution during various phases of contact with the ground. The gait phase, in accordance with the combination of the FSR sensor input, is shown in Figure 1. A single-board computer (SBC) with a Bluetooth module for wirelessly transmitting the data acquisition (DAQ) and gait phase recognition results from the 3-channel FSR array was developed to be strap-attachable to the ankle. Consequently, by utilizing the wireless FSR insole unit and IMU sensor developed in this study, we were able to gather the gait data of the subjects and label them effectively without any workspace limitations.

### 2.2. Sensor Calibration and Floating Body-Fixed Frame

**Figure 3:**
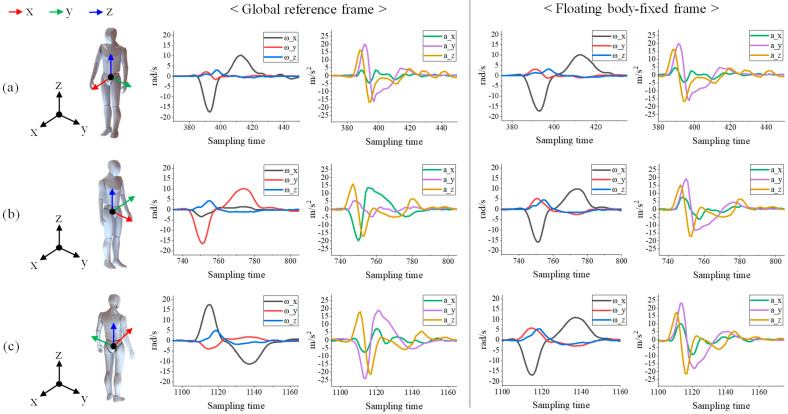
Sensor data output according to subject posture depending on whether the floating body-fixed frame is used or not. (**a**) The same pose as the initial reference frame, (**b**) rotate 90°, (**c**) rotate 180°.

The primary challenge in wearable Inertial Measurement Unit (IMU) sensor-based gait phase recognition research is the difficulty in maintaining consistent sensor orientation, as the attachment reproducibility of the sensor is low and the sensor fixed frame is expressed relative to a global reference frame, leading to orientation distortion with any deviation from the initial calibration pose. As shown in Figure 3 (left), even if the subject performs the same action while changing pose, the same pattern of data is not always collected, and the features that affect phase recognition change, resulting in low overall recognition accuracy. Therefore, this study solves the aforementioned problem by creating the float body-fixed frame (${FB}_{f}$), as proposed in previous our research [20].

**Figure 4:**
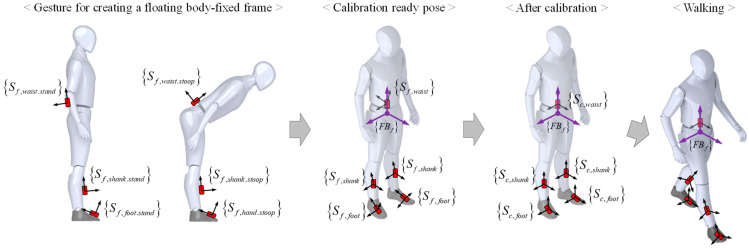
The procedure of creating a body-fixed frame and conducting sensor calibration.

Figure 4 illustrates the process of generating the ${FB}_{f}$ through a calibration gesture, followed by aligning the orientations of all IMU sensors to match the orientation of the newly created ${FB}_{f}$. Similar to Algorithm 1, the frontal horizontal axis can be determined through a stand–stoop operation, and the sagittal horizontal axis can be ascertained by computing the cross-product with the vertical axis, which remains parallel to the z-axis. In essence, a new reference frame, $B_{f}$, is established, aligning with the human body’s anatomical plane. To ensure that wearable IMU sensors, which may have varied orientations due to attachment variability, align with the orientation of the generated $B_{f}$, Algorithm 2 is employed. This algorithm calculates a rotation matrix that represents the orientation difference between the IMU sensor in the initial stand position and the $B_{f}$ orientation. It then adjusts the IMU sensors to match the pose of $B_{f}$. At this stage, the reference frame is modified such that the data output from the wearable IMU sensor, initially expressed in the global reference frame, is now represented from the newly established $B_{f}$. However, as $B_{f}$ is a fixed constant value within the global reference frame, it requires compensation to maintain alignment with the anatomical plane of the human body during movement. Therefore, as outlined in Algorithm 3, the orientation changes of the wearable IMU sensor attached to the waist are incorporated into the $B_{f}$, ensuring that the ${FB}_{f}$ consistently aligns with the human body’s anatomical plane, regardless of the person’s movement. In other words, the IMU sensor values are consistently represented relative to the new reference frame ${FB}_{f}$, which is aligned with the human body’s anatomical plane. This alignment ensures that the IMU sensor data remain consistent, even when the user changes posture or moves, as illustrated on the right of Figure 3. The formation and detailed information regarding the ${FB}_{f}$ are available in our previous research [20].
**Algorithm 1.** Create a body-fixed frame1:**procedure**
  sensor data RSf,jG,Ga→Sf,j,Gω→Sf,j
2:  **While** about 5 s3:    Maintain standing posture4:
    Save orientation data RSf,jG
5:  **end**
6:
  Update Rf,j.standG←avg(saved orientation data)
7:  **While** 5 s8:    Maintain stooping posture9:
    Save orientation data RSf,jG
10:
  **end**
11:
  Update Rf,j.stoopG←avg(saved orientation data)
12:
  **Calculate**
 vector k ←RTf,j.stoopG⋅Rf,j.standG
13:
  **Calculate**
 $\mathrm{vector}\;x\;\leftarrow \left(vector\;\;k\times {\left[\begin{array}{ccc} 0 & 0 & 1 \end{array}\right]}^{T}\right)$
14:
  **Return**
 $\mathrm{body-fixed}\;\mathrm{frame}\;\leftarrow \left[x_{B_{f}}\;\;\;k_{B_{f}}\;\;z_{B_{f}}\right]$


**Algorithm 2.** Align all sensor fixed frames equally1:**procedure** sensor data, body fixed frame, the orientation of the initial posture2:  **Calculate** rotated frame mapping 
  R C,jSf,j.stand=RTSf,j.standG⋅RBfG
3:
  **Calculate**
 $\mathrm{sensor}\;\mathrm{orientation}\;\mathrm{with}\;\mathrm{respect}\;\mathrm{to}\;\{B_{f}\}$

  RC,jBf=RTBfG⋅RSf,jG⋅R C,jSf,j.stand
4:
  **Calculate**
 $\mathrm{sensor}\;\mathrm{acceleration}\;\mathrm{with}\;\mathrm{resepect}\;\mathrm{to}\;\{B_{f}\}$

  a→Sf,shank/footBf=RTBfG⋅RSf,shank/footG⋅Ga→Sf,shank/foot
5:
  **Calculate**
 $\mathrm{sensor}\;\mathrm{rate}\;\mathrm{of}\;\mathrm{turn}\;\mathrm{with}\;\mathrm{respect}\;\mathrm{to}\;\{B_{f}\}$

  ω→Sf,shank/footBf=RTBfG⋅RSf,shank/footG⋅Gω→Sf,shank/foot
6:
  **Return**
 $\mathrm{sensor}\;\mathrm{data}\;\mathrm{with}\;\mathrm{respect}\;\mathrm{to}\;\{B_{f}\}$


**Algorithm 3.** Updating the body-fixed frame according to changes in subject’s time-varying body alignment1:**procedure** sensor data, the orientation of the initial posture2:  **Calculate** floating body fixed frame
  RFBfG=RSf,waistG⋅RSf,waist,standG
3:
  **Calculate**
 $\mathrm{sensor}\;\mathrm{orientation}\;\mathrm{with}\;\mathrm{respect}\;\mathrm{to}\;\{FB_{f}\}$

  RC,jFBf=RTFBfG⋅RSf,jG⋅R C,jSf,j.stand
4:
  **Calculate**
 $\mathrm{sensor}\;\mathrm{acceleration}\;\mathrm{with}\;\mathrm{respect}\;\mathrm{to}\;\{FB_{f}\}$

  a→Sf,shank/footFBf=RTFBfG⋅RSf,shank/footG⋅Ga→Sf,shank/foot
5:
  **Calculate**
 $\mathrm{sensor}\;\mathrm{rate}\;\mathrm{of}\;\mathrm{turn}\;\mathrm{with}\;\mathrm{respect}\;\mathrm{to}\;\{FB_{f}\}$

  ω→Sf,shank/footFBf=RTFBfG⋅RSf,shank/footG⋅Gω→Sf,shank/foot
6:
  **Return**
 $\mathrm{sensor}\;\mathrm{data}\;\mathrm{with}\;\mathrm{respect}\;\mathrm{to}\;\{FB_{f}\}$


### 2.3. Data Acquisition and Preprocessing

#### 2.3.1. Data Acquisition

**Figure 5:**
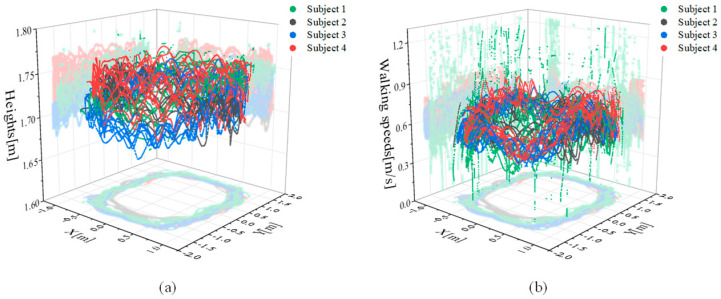
Subject-specific gait trajectories measured with an OptiTrack motion capture camera for data acquisition. (**a**) Walking trajectory, (**b**) walking speed.

In this study, to collect labeled walking data, four subjects with diverse physical conditions (as detailed in Table 1) had markers attached to their heads for measuring walking trajectories. They walked at speeds ranging from 0.24 to 1.37 m/s on a 4 × 4 m² flat surface, where a Prime13 motion capture camera (OptiTrack, Corvallis, OR, USA) was installed, changing walking directions, as depicted in Figure 5. We collected approximately 29,000 data points, comprising orientation, acceleration, and angular rate, while subjects freely changed direction. Approximately 24,000 data points from three of the four subjects (subjects 1, 2, and 3) served as training datasets for developing prediction models, while around 5000 data points from the remaining subject (subject 4) were utilized as a test dataset to validate the learned model.

#### 2.3.2. Data Augmentation

A large amount of data is required to improve the performance of recognition models and prevent overfitting. Due to the difficulty in achieving high accuracy and generalization performance with the initially collected 24,000 training data, data augmentation is performed. Data augmentation of the gait data collected from IMU sensors, which are inherently time-series data, must be carried out to preserve their temporal dependency feature. There are various methods available for augmenting time-series data. Among these, data augmentation techniques applied in the time domain are considered the most practical and widely used [24]. Among the time domain techniques, the noise injection method that adds Gaussian noise is similar to the sensor noise that occurs when collecting IMU sensor data, so the noise injection method is used in this study. As described in Equation (1), data augmentation, in this study, is executed by adding Gaussian noise $W\left[n\right]$ to each feature of the labeled IMU gait data $S\left[N\right]$. This Gaussian noise is of a magnitude equivalent to 10% of ±(max(|feature data|) − |mean|), ensuring the addition aligns with the distribution characteristics of the original sensor data. Through this process, approximately 24,000 training data points were augmented to a total of 359,924, achieving an augmentation increase of about 15 times.

(1) $$S\left[N\right]+W\left[N\right]=N\left[n\right]$$

#### 2.3.3. Standardization

**Figure 6:**
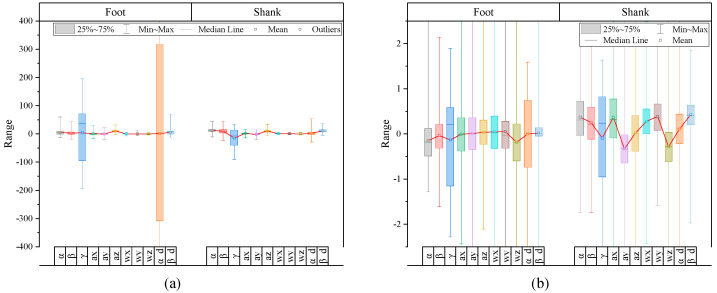
Distribution range of gait data features. (**a**) Before performing standardization, (**b**) after performing standardization.

Figure 6a is the result of plotting the gait data collected from IMU sensors attached to each lower limb segment for each feature before standardization. As seen in the figure, each feature has a considerable scale difference. When learning a recognition model with feature data showing such a significant difference, features with a wide distribution of data mainly affect learning, and features with a small distribution do not significantly impact learning. In other words, recognition accuracy decreases because the number of features that affect gait phase recognition falls. Therefore, standardization was performed through Equations (2) and (3) so that all feature data had a similar scale.

(2) $$\sigma_{j}=\sqrt{\frac{\sum_{i=1}^{N}{\left(x_{i,j}-E\left(x_{j}\right)\right)}^{2}}{N}}$$

(3) $${\tilde{x}}_{i,j}=\frac{\left(x_{i,j}-{\bar{x}}_{j}\right)}{\sigma_{j}}$$

Here, i corresponds to the time stamp, and j represents each feature column. $x_{i,j}$ is raw feature data, $E\left(x_{j}\right)$ is the mean value of feature $x_{j}$, and $\sigma_{j}$ and ${\tilde{x}}_{i,j}$ are the standard deviation and normalization results of feature data $x_{j}$, respectively. After standardization, as shown in Figure 6b, the influence of the scale of specific feature data was significantly reduced, and the distribution pattern of features between labels was clearly visible, which is expected to be a positive factor in learning a multi-gait phase recognition model.

#### 2.3.4. Sliding Window Label Overlapping Method

**Figure 7:**
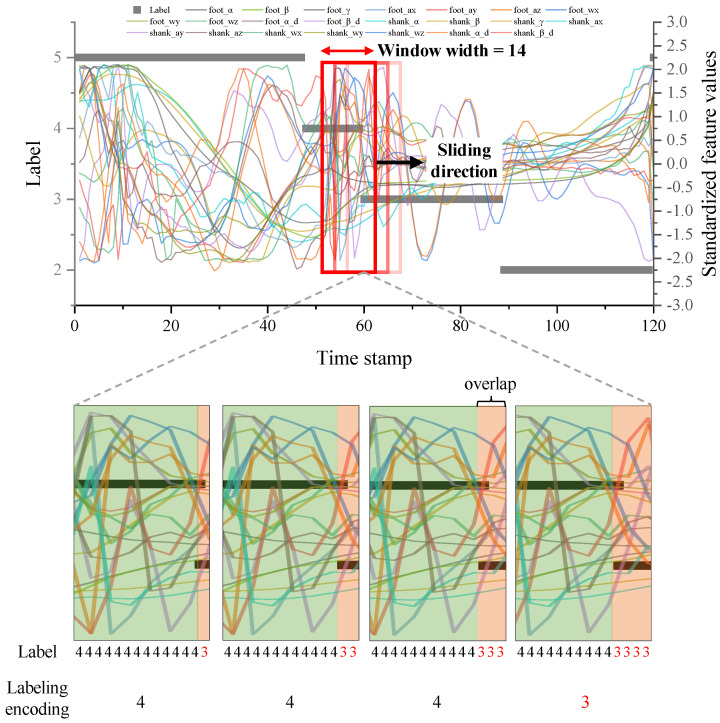
Generation and labeling of training data using the sliding window overlapping method.

Bi-directional LSTM, which is used as a gait phase recognition model in this study, mainly uses the sliding window method to continuously extract and predict data features in every cycle [25]. However, if label overlapping is allowed in time-series data where the ordered label appears repeatedly, two labels are included in the sliding window where the labels transition, as shown in Figure 7. Therefore, labeling these sliding windows is also important. In this study, we aim to estimate the current phase by referring to past data through a window with an experimentally found optimal window size of 22 (height: feature) × 14 (width: time) when the last N of the labels of the sliding window are the same, it is encoded into the label of the sliding window. At this time, how many of the last N labels are included in the 14-time horizons and whether label encoding is performed also significantly impacts the prediction model recognition accuracy. Therefore, we divide the label overlapping ratio by 30%, 50%, and 70% to select the optimal parameter through experiments in the next section.

### 2.4. Bidirectional LSTM-Based Gait Phase Recognition Model

**Figure 8:**
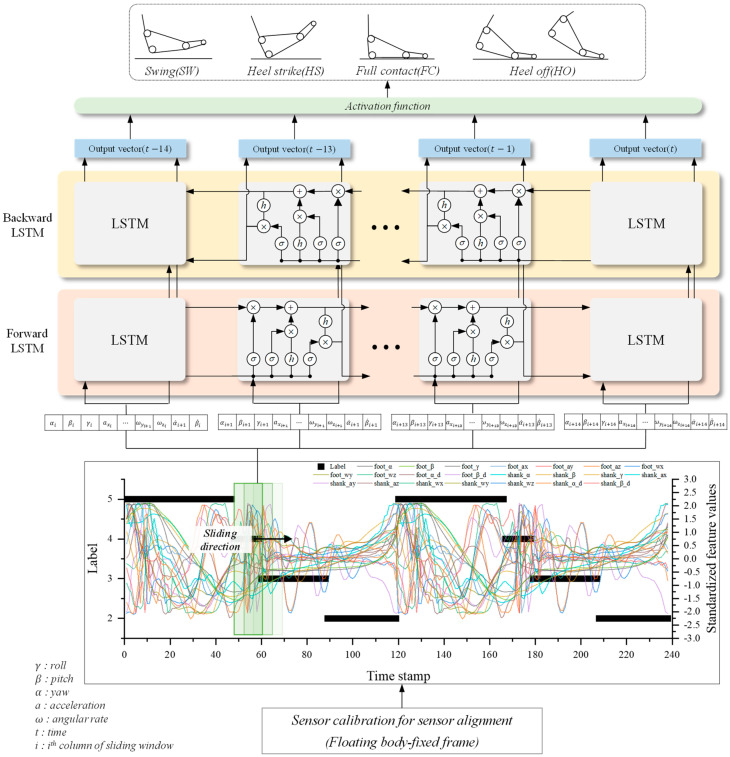
Structure of the bi-directional LSTM-based gait phase recognition model.

In this study, we used bi-directional LSTM based on previous research showing that bi-directional LSTM is capable of recognizing not only the instantaneous mode of repetitive and regular motion but also the long-term dependency between modes [20], and the detailed model structure is shown in Figure 8. The 22 gait data for each lower extremity segment consisting of orientation, acceleration, and angular rate collected through the FBF method and IMU are captured at 1 ms intervals through standardization and a sliding window overlapping method with a window of 22×14 size. The captured 22×14 data goes through a forward LSTM model and a backward LSTM model to identify the walking context. Afterward, the output hidden vector is predicted to be one of four gait phases through the activation function.

## 3. Experiment and Conclusions

### 3.1. Sensitivity Analysis

The selection and setting of parameters and hyperparameters in training deep learning models significantly impact both learning and test accuracy [26,27]. Therefore, it is important to select the parameters that influence model training and find their optimal values. In this study, eight parameters were selected as key design variables to maximize the accuracy of gait phase recognition. The activation functions chosen were tanh, selected for its ability to appropriately model nonlinear data characteristics and provide a suitable output range for LSTM gates, and LeakyReLU, utilized to mitigate the issue of gradient vanishing. The overlapping ratio was set to 30%, 50%, and 70% according to the number of the last N labels, as described in the previous section. The number of layers was selected in consideration of the model’s complexity and computational efficiency, and the optimizer was selected as Adam, Nadam, and AdamW to increase convergence efficiency in model learning. The size of the hidden units and the learning rate were considered crucial factors for determining the model’s ability to process complex data and the speed of efficient learning, respectively. Thus, they were set within an appropriate range. The dropout ratio was adopted at an appropriate level to prevent overfitting and enhance the model’s generalization ability. Finally, the batch size was chosen considering both the stability of gradient descent and memory efficiency. These parameters are vital in optimizing the performance of the model, and the level for the parameter was selected, as shown in Table 2, to find the optimal combination through level average analysis of the Taguchi method $L_{18}\left(2^{1}\times 3^{7}\right)$.

**Figure 9:**
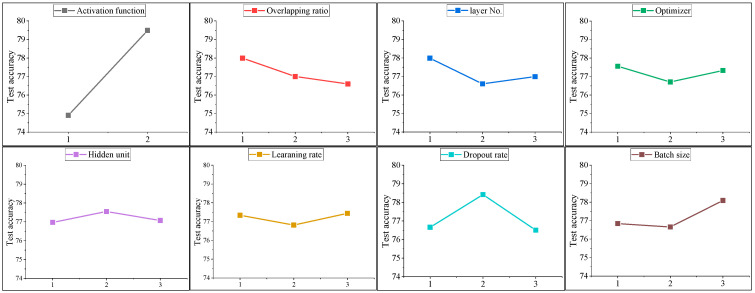
Result of the level average analysis.

In the experiment, about 360,000 data collected from three subjects were divided into a training dataset and a validation dataset at a 6:4 ratio, and bi-directional LSTM model learning was conducted according to the combination of 18 design variables in Table 3. Additionally, the test accuracy of the learned model was confirmed with about 5000 data collected from one other subject that was not used for learning. Figure 9 presents the sensitivity analysis result for the test accuracy of the learned model, and Table 4 outlines the optimal parameter set derived through sensitivity analysis. In the case of the activation function, LeakyReLU showed significantly higher test accuracy than tanh, which has greatly contributed to LeakyReLU’s ability to solve the vanishing gradient problem. The overlapping ratio showed the highest test accuracy when it was 30%, and a single layer was found to be the most efficient. This is expected to be because gait phase data have a simple characteristic of repeated cycles, so a deeper architecture may actually cause overfitting. The optimizer analysis demonstrates that basic Adam efficiently finds the most effective convergence path. For hidden units, selecting 64 optimizes the balance between model complexity and overfitting avoidance, allowing for accurate gait data modeling without excessive computational load. In the case of the learning rate, it was confirmed that 0.003 is the optimal value, which indicates that it can converge faster while maintaining stability. In the case of the dropout rate, it was confirmed that 0.5 is the most optimal value. This means that significant normalization seems necessary, as even with simple, repetitive phase gait data, the risk of overfitting to the gait patterns of the three subjects used for training can severely impair the generalization performance. Finally, in the case of batch size, 7000 was found to be the most optimal value, which shows that the global minimum can be found stably based on a lot of data.

### 3.2. Optimal Parameter Learning Result and Conclusions

**Figure 10:**
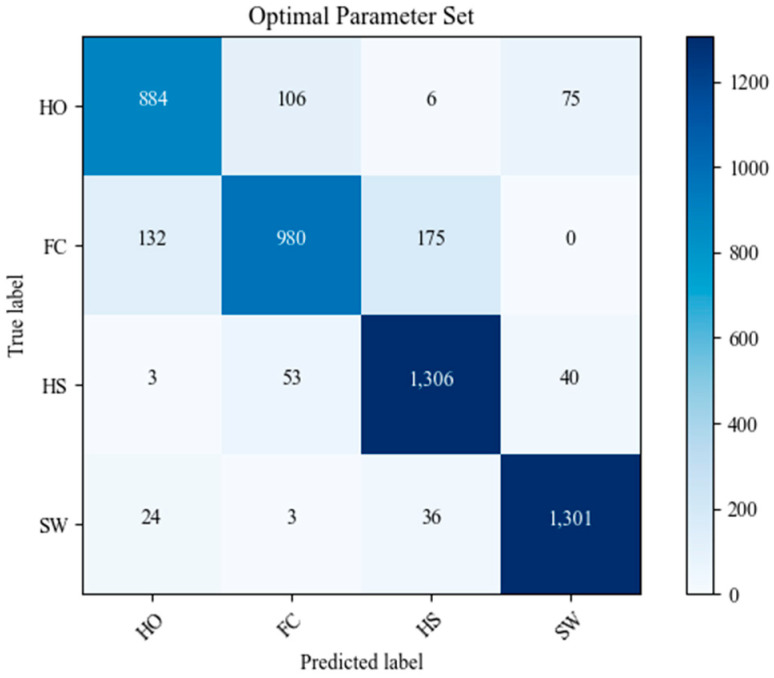
Confusion matrix of the prediction results for the test dataset.

The bi-directional LSTM model, coded in Python 3.11 and TensorFlow 2.14.0, with optimal parameters, was trained and tested for gait phase prediction on a computer equipped with an AMD Ryzen 5950 × 4th generation CPU and a 3090 GPU. As a result, the model achieved a high test accuracy of 86.43%. This represents an improvement of approximately 122% over the lowest test accuracy shown by set 5 in the orthogonal array combinations and about 105% over the highest test accuracy demonstrated by set 15. Table 5 shows that the SW phase test accuracy was the highest at 91.39%. This was followed by a nearly uniform accuracy across the remaining stance phases: FC at 85.45%, HS at 85.08%, and HO at 83.82%. Figure 10 shows the confusion matrix of the prediction results. In the figure, SW and HS show high true positive rates in the diagonal values, while FC and HO frequently exhibit adjacent off-diagonal values. This can be attributed to two main factors. Firstly, as seen in the left walking data of Figure 7, most of the IMU sensor features show little change in values during the transition from label 3 (FC) to label 2 (HO), making it difficult to distinguish between these labels. Additionally, this study uses a sliding window method that allows for label overlapping, contributing to the tendency of misclassifying between FC and HO. Therefore, future research is needed to improve accuracy in the transition sections of ordered time-series data when predicting phases using the sliding window label overlapping method, particularly where there is a lack of features to distinguish phases.

## Figures and Tables

**Table 1:** Detailed physical information of the participants in the experiment.

Subject	Gender	Height [cm]	Weight [kg]	Foot Size [mm]
1	Man	177	100	270
2	Man	171	73	260
3	Man	172	88	265
4	Man	179.9	73	270

**Table 2:** Design parameters and level for each parameter.

Level	Activation Function	Overlapping Ratio [%]	Layer No.	Optimizer	Hidden Unit	Learning Rate	Dropout Rate	Batch Size
1	Tanh	30	1	Adam	32	0.001	0.3	3000
2	LeakyReLU	50	2	Nadam	64	0.002	0.5	5000
3		70	3	AdamW	128	0.003	0.7	7000

**Table 3:** Orthogonal array table.

Set	Activation Function	Overlap Ratio [%]	Layer No.	Optimizer	Hidden Unit	Learning Rate	Dropout Rate	Batch Size	Test Accuracy
1	1	1	1	1	1	1	1	1	76.36
2	1	1	2	2	2	2	2	2	75.76
3	1	1	3	3	3	3	3	3	76.43
4	1	2	1	1	2	2	3	3	74.29
5	1	2	2	2	3	3	1	1	71.10
6	1	2	3	3	1	1	2	2	73.49
7	1	3	1	2	1	3	2	3	78.01
8	1	3	2	3	2	1	3	1	74.55
9	1	3	3	1	3	2	1	2	74.15
10	2	1	1	3	3	2	2	1	81.08
11	2	1	2	1	1	3	3	2	78.35
12	2	1	3	2	2	1	1	3	79.95
13	2	2	1	2	3	1	3	2	80.13
14	2	2	2	3	1	2	1	3	80.33
15	2	2	3	1	2	3	2	1	82.66
16	2	3	1	3	2	3	1	2	78.07
17	2	3	2	1	3	1	2	3	79.53
18	2	3	3	2	1	2	3	1	75.28

**Table 4:** Optimal design parameter combination.

Activation Function	Overlapping Ratio [%]	Layer No.	Optimizer	Hidden Unit	Learning Rate	Dropout Rate	Batch Size
LeakyReLU	30	1	Adam	64	0.003	0.5	7000

**Table 5:** Test accuracy for each gait phase.

	SW	HS	FC	HO	Total
Accuracy [%]	91.39	85.08	85.45	83.82	86.43

## Data Availability

The data used to support the findings of this study are available from the corresponding author upon request.

## Nomenclature

R	Rotation matrix
$\left\{G\right\}$	Global reference frame
$\left\{B_{f}\right\}$	Body-fixed frame
$\left\{FB_{f}\right\}$	Floating body-fixed frame
$\left\{S_{f,j}\right\}$	Sensor-fixed frame of *j*th IMU sensor
$\left\{C_{j}\right\}$	Calibrated sensor-fixed frame
$\left\{S_{f,j.stand}\right\}$	Sensor-fixed frame at initial standing posture
$\left\{S_{f,j.stoop}\right\}$	Sensor-fixed frame at initial stooping posture
$\vec{a}$	Acceleration
$\vec{\omega }$	Angular rate
